# Embryo donation among Latin-Americans who have attended assisted reproduction techniques: a first empirical approach

**DOI:** 10.5935/1518-0557.20200055

**Published:** 2021

**Authors:** Jorge Alberto Álvarez-Díaz

**Affiliations:** 1 Departamento de Atención a la Salud. Universidad Autónoma Metropolitana. Unidad Xochimilco, México

**Keywords:** embryo transfer, embryo destination, embryo research, reproductive techniques, bioethics

## Abstract

**Objective:**

To determine whether Latin Americans who have undergone assisted reproduction techniques would donate embryos.

**Methods:**

This is a multinational cross-sectional study, involving 602 patients. We invited the Latin American Network of Assisted Reproduction centers. Those who accepted received the instrument distributed among the patients who agreed by signing the informed consent form. In total, 261 men and 341 women participated from seven countries.

**Results:**

Patients would donate their embryos as follows: treatment with embryonic stem cells (73.6%), heterosexual couples (63.8%), Assisted Reproduction Techniques (ART) research (57%), scientific or basic research (55.3%), research with embryonic stem cells (55.2%), premenopausal women (53.8%), single women (45.1%), people with disabilities (25.4%), lesbians (25.3%), menopausal women (25.2%), lesbian couples (24.6%), gay couples (19.6%), senile women (15.1%).

**Conclusions:**

The results favor embryos donation for research purposes, and a little less for clinical purposes, contrary to what was thought in qualitative studies conducted among Latin American populations.

## INTRODUCTION

The first in vitro fertilization occurred in a physiological cycle. Techno-scientific approaches hyperstimulated the ovary so that it produced more than one oocyte, thus increasing the odds of achieving fertilization and implantation, and eventually a live newborn. Therefore, new medical possibilities and bioethical issues emerged: oocyte donation, embryo donation, and the possibility of transferring embryos to a different woman than the one who produced the gamete (surrogacy).

We estimate the need for approximately fifteen oocytes to achieve a live birth (Sunkara *et al*., 2011). While obstetric history must be considered clinically (Goldberg *et al*., 2016); the current trend is the transfer of a single embryo. The development of vitrification as a cryobiology technique for the preservation of embryos in a blastocyst reduced the number of oocytes captured and the number of embryos generated (Liebermann, 2017).

When the “leftover embryos” appeared, they were given several names that do not necessarily express the same meaning: remnants, supernumeraries, surpluses, leftovers, spare, unused, unwanted, renounced, unclaimed and abandoned. Alternatively, the term “embryo donation” became “embryo adoption”, common in the Christian tradition (Robertson, 2018). According to the American Society for Reproductive Medicine (ASRM), its use is inaccurate and not recommended (Ethics Committee of the ASRM, 2016a). In this study, we used the most accurate concepts of “remaining embryos” and “embryo donation” (ED). The Latin American Registry of Assisted Reproduction (RLA) of the Latin American Network of Assisted Reproduction (REDLARA) only includes the number of embryonic transfers and embryo transfers originated through oocyte donation, but it does not have data on ED, so that it is challenging to have quantitative approximations of the phenomenon among Latin Americans (Zegers-Hochschild *et al*., 2019).

ED has reproductive and non-reproductive indications (Dayal, 2013). Among the first are couples with poor prognosis indicators to achieve pregnancy (ovarian failure, tubal factor, uterine factor, male factor, carriers of genetic diseases, cancer survivors, failure in previous attempts with assisted reproduction techniques (ART), and the like). Among the latter, the economic factor prevailed, since if women only go for embryo transfer, the costs decrease by at least half; ED is approximately twice as profitable as donating oocytes, concerning cost per live birth (Finger *et al*., 2010), and even cheaper than adopting a child (Gilman & Freivalds, 1997). ED also provides single women and lesbian couples with an additional opportunity to have children of their own (Marina *et al*., 2010). Another non-reproductive indication has been research.

Theoretically, there are three possible destinations for remaining fresh embryos: cryopreserve, discard and donate. Three possible destinations are also available for the remaining cryopreserved embryos: remaining cryopreserved for own use, discarded and donated. Therefore, at least two moments are at hand to decide the fate of the remaining embryos: fresh and cryopreserved. Finally, couples donate their embryos for the following possible purposes: clinical use (or reproductive use, own use, or for someone else), research, and education. ED for education (training of clinical embryologists) is a proposal, even in developed countries.

Research with human embryos generated a growing literature on embryonic stem cells. In 1998, a group from the University of Wisconsin isolated cells from the internal embryonic cell mass and developed the first embryonic stem cell lines (Thomson *et al*., 1998). In Latin America, the first line of human embryonic stem cells came from Colombia in 2006 (Lucena *et al*., 2006). Although research on induced pluripotent stem cells has grown, generating them does not solve the problem of remaining cryopreserved embryos, since they were generated in parallel during the process of assisted reproduction.

This study aimed to investigate the opinions for ED in a sample of infertile Latin American patients in order to obtain information that could be useful for reproductive medicine centers and the design of ED standards, regulations, or policies (both ethical and legal), without violating the preferences of couples.

## MATERIALS AND METHODS

We contacted and invited REDLARA accredited centers. Those who responded received complete information about the study (project and instrument). Some members of the reproductive medicine center team made the personal invitation to participate and carried out the informed consent process. If patients agreed to participate in the investigation, they would answer the questionnaire themselves, and after doing so, they could withdraw their consent, not submitting the completed questionnaire. The questionnaire was anonymous in order to preserve confidentiality.

This is a cross-sectional, observational, and descriptive study. We created an ad hoc instrument, consisting of questions used in previous similar surveys found in the background of this study and relevant for the study, and many other questions were explicitly created. The first part investigates personal and sociodemographic background. The second part explores religious beliefs and practices. The third part includes very brief information on their knowledge about medical history. The fourth part consisted of opinions towards ED, divided into four subgroups: the feeling of parenthood, ED for clinical purposes, ED for research purposes, and the importance of genetic linkage. We used a Likert scale to respond to the statements contained in the fourth part, where 1 represents “totally agree”, 2 “moderately agree”, 3 “neither agree nor disagree”, 4 “moderately disagree” and 5 “totally disagree”. The questionnaire had two versions (women and men). For Brazil, we used a certified translation reviewed by assisted reproduction professionals. The questionnaire also had information about the study and contact details of the principal investigator.

The inclusion criteria included: being Latin American by birth, living in a Latin American country, having already lived at least one cycle with any ART (with or without pregnancy), and agreeing to sign the informed consent form. There were no exclusion criteria. The elimination criteria were: being Latin American by birth but living outside the Latin American region, not being Latin American by birth but living in Latin America, and submitting an incomplete questionnaire.

The survey registration, statistical processing, and analysis were performed with SPSS version 16. The study was approved first at the Complutense University of Madrid, then by the REDLARA Bioethics Interest Group, and finally at each center that agreed to participate. We considered ethical aspects of the Helsinki Declaration and the CIOMS Guidelines, because of the international nature of this study.

## RESULTS

In total, 702 patients agreed to participate as research subjects, agreeing to answer the questionnaire. Once completed, 18 refused to submit it, and 25 of the remaining ones were eliminated because the respondents were Latin Americans living in a developed country, 12 were not Latin Americans (in “reproductive tourism” or “cross-border reproductive care”), and 45 did not respond the questionnaire completely. Thus, we arrived at 602 research subjects (261 men and 341 women), from 15 reproductive medicine centers in 9 cities (Quito, Salto, La Paz, Lima, Bogotá, Caracas, Valencia, Ribeirão Preto, and São Paulo) and seven countries (Ecuador, Uruguay, Bolivia, Peru, Colombia, Venezuela, and Brazil).

Table 1 below summarizes the most relevant sociodemographic characteristics of the respondents (Table 1). Their mean age was 37.4 years, with a standard deviation of 5.7; 92% were legally married; 67.8% had no children; 34.9% had a bachelor’s degree, and 25.1% had postgraduate education; 89% identified themselves as part of a religion; of those who felt part of a religion, 81.7% assumed to belong to the Catholic Church. Only 31.7% of the participants responded that they had cryopreserved embryos. The mean response cycle was 2.2, with a standard deviation of 1.4.

**Table 1:** Sociodemographic data.

Sociodemographic data.		Men	Women
Marital status	Single Married Divorced Widower Other	4 246 4 1 6	17 308 5 1 10
Having children	No Yes, from previous couple Yes, of current couple Yes adopted Yes, with previous partner and current partner	167 42 47 4 1	241 23 70 5 2
Scholarship	Undergraduate Bachelor or equivalent Master or equivalent Doctorate or equivalent Unspecified Postgraduate Postdoctoral	111 88 6 4 50 2	130 122 19 2 68 0
Country	Ecuador Uruguay Bolivia Peru Colombia Venezuela Brazil	3 7 9 12 15 80 135	4 7 11 27 38 102 152
Religion	Any Catholic Christian Non-Catholic Christian Buddhist Bean Muslim Masonry Santeria Theist without religion ascription Spiritism	32 178 29 1 3 1 1 1 1 14	27 255 37 0 1 0 0 0 0 21
Known fertility problems	Male factors Female factors Combined factors	97 102 62	116 135 90
TRA	IVF ICSI IVF & ICSI IVF with egg donation FIV with egg donation IMSI	112 119 15 1 0 14	144 143 36 4 1 13
Transfer procedures	1 2 3 4 5 6 7 8	103 79 42 23 7 5 1 1	129 91 70 30 8 8 2 3
Cryopreserved embryos	Yes No	83 178	108 233

We assessed their feeling of parenthood, that is, the moment in which the participants identified themselves as mothers or fathers. This point provides data on how they understood the status of the embryo. Qualitative data published before this research showed that infertile couples do not have the same considerations about embryo status. They frequently refer to embryos as “children”, even when they are cryopreserved. On the other hand, particularly when they have had negative experiences (no implantation and therefore no pregnancy; spontaneous abortion, and the like), the patients were identified as fathers or mothers at very different times of the assisted reproduction cycle: fertilization (in some centers, biologists comment on how many oocytes were recovered and how many of them were fertilized, and there are even those who allow parents to observe them under the microscope), embryo transfer, with positive pregnancy test, ending the first trimester of pregnancy (in some countries, even fertile women wait for this period to announce the pregnancy to family and friends), until they could see abdominal growth, and those who identified themselves as father or mother later mentioned the birth. No one referred to it after birth. More than half (60.1%) of the research subjects were considered as parents when they received a positive pregnancy test (Table 2).

**Table 2:** Feeling of parenthood

Feeling of parenthood		Men	Women
From fertilization	I don't know; does not apply Totally agree In agreement In disagreement Strongly disagree	55 37 63 75 31	66 52 65 122 36
From embryo transfer	I don't know; does not apply Totally agree In agreement In disagreement Strongly disagree	47 63 86 47 18	62 93 114 54 18
From the positive pregnancy test	I don't know; does not apply Totally agree In agreement In disagreement Strongly disagree	85 93 58 13 12	108 157 54 15 7
From the end of the first trimester of pregnancy	I don't know; does not apply Totally agree In agreement In disagreement Strongly disagree	103 69 45 25 19	130 113 45 30 23
From the beginning of abdominal growth	I don't know; does not apply Totally agree In agreement In disagreement Strongly disagree	111 75 37 21 17	135 112 46 28 20
From birth	I don't know; does not apply Totally agree In agreement In disagreement Strongly disagree	106 106 18 18 13	144 129 23 23 22

The next section of the instrument analyzes the opinions on ED for clinical purposes (Table 3). The distribution of participants donating their embryos was as follows: 63.8% to heterosexual couples; 53.8% to premenopausal women; 45.1% to single women; 25.4% to people with disabilities, 25.3% to lesbians; 25.2% to menopausal women; 24.6% to lesbian couples; 19.6% to gay couples, and 15.1% to older women.

**Table 3:** Embryo donation for clinical purposes

Embryo donation for clinical purposes		Men	Women
Heterosexual couple	I don't know; does not apply Totally agree In agreement In disagreement Strongly disagree	42 57 109 31 22	51 73 145 41 31
Single woman	I don't know; does not apply Totally agree In agreement In disagreement Strongly disagree	49 34 78 67 33	54 50 109 81 47
Lesbian woman	I don't know; does not apply Totally agree In agreement In disagreement Strongly disagree	42 16 52 78 73	60 20 64 98 99
Woman in pre-menopause	I don't know; does not apply Totally agree In agreement In disagreement Strongly disagree	48 35 99 46 33	55 50 140 57 39
Woman in the climacteric	I don't know; does not apply Totally agree In agreement In disagreement Strongly disagree	71 27 50 82 31	92 22 53 127 47
Woman in post-menopause	I don't know; does not apply Totally agree In agreement In disagreement Strongly disagree	56 22 31 87 65	82 14 24 121 100
People with disabilities	I don't know; does not apply Totally agree In agreement In disagreement Strongly disagree	79 22 52 64 44	108 20 59 95 59
Lesbian couple	I don't know; does not apply Totally agree In agreement In disagreement Strongly disagree	39 15 48 79 80	48 18 67 109 99
Gay couple	I don't know; does not apply Totally agree In agreement In disagreement Strongly disagree	35 11 33 75 107	42 13 61 113 112

The next section of the instrument analyzes the opinions on ED for research purposes (Table 4). Participants would donate embryos as follows: 73.6% for stem cell treatments; 57% for ART research; 55.3% for general scientific research; 55.2% for general scientific research; 55.2% for stem cell research; and 55.1% for research that is supervised to ensure it is carried out for the purposes for which they consented.

**Table 4:** Embryo donation for research purposes

Embryo donation for research purposes		Men	Women
For scientific research in general (basic science)	I don't know; does not apply Totally agree In agreement In disagreement Strongly disagree I don't know; does not apply	46 59 83 50 23 47	49 74 117 72 29 54
For research with personal verification	Totally agree In agreement In disagreement Strongly disagree	45 94 53 22	59 134 71 23
For research in reproductive medicine	I don't know; does not apply Totally agree In agreement In disagreement Strongly disagree	43 43 100 53 22	43 58 142 79 19
Stem cell research	I don't know; does not apply Totally agree In agreement In disagreement Strongly disagree	53 36 97 52 23	50 45 154 73 19
Stem cell treatment	I don't know; does not apply Totally agree In agreement In disagreement Strongly disagree	47 64 118 21 11	41 96 165 28 11

## DISCUSSION

The issue of embryo status has generated heated debates. Much has been said that it could be related to religiosity, citing the “Artavia Murillo y otros. (In vitro fertilization) *vs.* Costa Rica” case of the Inter-American Court of Human Rights. The only confessional Central American country, Costa Rica, banned ARTs. The Court considered that it had violated the human rights of those who sued the Costa Rican state so that it was requested to pay for damages (Inter-American Court of Human Rights, 2012). The ruling is also cited in the Province of Buenos Aires, where they recognize “every human being from the moment of conception” as a “child” (Cámara Nacional de Apelaciones en lo Civil, 1999). Argentinian law used the term “dación de embriones” (from “given embryo”). However, it is not a purely Latin American issue. In France, one speaks of “l’accueil d’embryon”, which can be translated as “embryo reception”. However, the term “accueil” also means “welcoming”, and they do not use the term “donner” (meaning “donate”; there are no references to the term “don d’embryon”, which would be the translation to “embryo donation”). This has been interpreted as an “ultra-early form of adoption” (Malzac, 2011). Besides the ASRM, the European Society for Human Reproduction and Embryology (ESHRE) also uses and recommends the term “embryo donation” instead of “embryo adoption” (ESHRE Task Force on Ethics and Law, 2002).

Despite these and other arguments, the empirical data is inconclusive. Some studies show an association between religiosity and the possibility of donating embryos (Mohler-Kuo *et al*., 2009), unlike some other studies (Bangsbøll *et al*., 2004). There was no statistical association between ED and religiosity in this study. One possible explanation may be the “popular use of religion” (Rostas & Droogers, 1993). According to these analyses, religious people express the official position of the Church (mainly Catholic, which does not officially accept ART; Vatican, 1995), but reinterpreted with their personal experiences, so they may feel that “God is in the laboratory” (Roberts, 2006).

The Latin American constant in legal regulation on the subject is the void. However, it does not also mean that people would not agree to any kind of legal regulation in place. An example is Sweden, where embryos can be cryopreserved for five years and, if not claimed, discarded. While ED is not allowed, research shows that both reproductive medicine professionals (Wånggren *et al.,* 2014) and the general population (Wånggren *et al*., 2013a; b) would agree with ED. Another example is Germany, where it is forbidden to generate remaining embryos, donate them, and do research with them; however, while patients, geneticists and gynecologists would approve these practices, ethicists would not (Krones *et al*., 2006).

Opinions about ED for clinical or reproductive purposes show that between one-fifth and two-thirds are in favor of donating to the different contexts raised in the instrument. Data in developed countries show that up to 75% agree to donate for reproductive purposes (Wånggren *et al*., 2013a; b). One possible explanation is that respondents have their own experiences, knowledge, and understanding of various ART possibilities and are more positive about ED, to help other infertile couples in their effort to have children (the same project the participants of this research have or had). Some of the respondents in this study have been successful with the help of ART, while others have not, and perhaps this positively affects the response rate, acceptance, and altruistic motive. Some research has shown that the desire to help others could be significant in ED (McMahon *et al*., 2003). It is also notable that those who donate embryos or those who receive them do not see the procedure as similar to adoption (Millbank *et al*., 2017).

The least favored option in this investigation was ED to older women, where the women pregnancy age limit is a controversial issue. The ARTs allowed postmenopausal women to take on a pregnancy (with oocyte or embryo donation plus hormonal support). However, the possibility of obstetric complications increases with advanced maternal age, so that health, in general, and cardiovascular health, in particular, should be carefully evaluated (MacArthur *et al.,* 2016). The ASRM recommends deterring women over 55 years of age seeking pregnancy, due to possible complications (Ethics Committee of the ASRM, 2016b).

On the other hand, between a fifth and a quarter of the participants would donate embryos to gay and lesbian couples. This research shows that if the ED is for single women, almost half of the respondents would donate embryos, but it falls to half (a quarter) if the recipients are lesbian. Respondents must consider the possibility of donating embryos for non-heterosexual people, even if in a low proportion. The LGBT community is another face of ARTs. The ASRM considers that it is unethical to prevent access to infertility treatments for single, gay, or lesbian people (Ethics Committee of ASRM, 2013). ESHRE extends it to gay and lesbian couples and transgender people (De Wert *et al*., 2014). This data show that some Latin Americans share liberal and non-discriminatory opinions, such as those of ASRM and ESHRE. ARTs have increased the classic anthropological discussions about kinship: family, father, mother, and the like are concepts that have historical and sociocultural constructions (Álvarez-Díaz, 2014). There are some recent Latin American studies, which analyze the outcome of children raised by LGTBI parents. It would be useful to educate patients about this issue, diminish fears and promote understanding (Zegers & Salas, 2014).

Among the most favored options are those associated with research purposes, especially ED, to achieve stem cell treatments. A systematic review shows that donating embryos specifically for research ranges from 7% in France to 73% in Switzerland (Samorinha *et al*., 2014). Those who wish to donate embryos usually describe that decision as better than discarding them; also, they feel reciprocity towards science and medicine, since they see themselves as recipients of their benefits by having been able to go to an ART. The review shows that the most important reasons not to donate were the perception of the risks, conceptualizing the embryo as a person, and lack of information about the research projects. In this investigation, if the participants knew the project and had a way to verify it, their response was very favorable. If we are looking to build public policies and not merely state policies, we must consult the opinions and suggestions of citizens, where potential donors and reproductive medicine professionals stand out (Samorinha & Silva, 2016).

The investigation had some limitations. One inherent to reproductive medicine services in Latin America, because most centers are private, introducing sociodemographic biases in the sample (economic and educational). Some other particular limitations are related to areas not included in the instrument used: age of receiving men, trans people, whether the donation should be anonymous or open, and the like. The ASRM suggests that the offspring should be informed about their origin (Ethics Committee of the ASRM, 2018). Advice to donors, recipients, their families, and the offspring is a crucial issue (Goedeke *et al*., 2016). The data shows that reproductive medicine professionals can instruct their patients well on the process of a common ART, but little information is managed regarding the issue of ED (Deniz *et al*., 2016).

On the other hand, Latin American countries that consider regulation should have a general legal framework on ARTs, and something specific that considers ED in particular. From a purely biomedical perspective, ED is a simple procedure (it involves embryonic transfer). The difficulty lies mainly in its complex ethical and legal aspects. It is known, for example, that disputes in cases of divorce or death of any of the couple members have led to lengthy legal proceedings, among other things, due to the absence of adequate laws (Cohen & Adashi, 2016). Some countries restrict or prohibit ED practices, such as Brazil, Denmark, Israel, Japan, Norway, Sweden, Switzerland, Taiwan, and Turkey. In other nations, ED is possible under various conditions, such as in Germany, Australia, Austria, Canada, Spain, U.S., Finland, France, Italy, New Zealand, and the United Kingdom. The donation practice form also varies substantially, from anonymous, which has historically been the most common form of donation, and is allowed in Bulgaria, Denmark, Spain, Greece, India, Portugal, the Czech Republic, and South Africa; and allowed with varying degrees of open donation. On the other hand, Canada, Norway, New Zealand, the Netherlands, the United Kingdom, Sweden, and some states of Australia allow donations in the context of donor identity registration, where those conceived may access a donation program to know their genetic information (Goedeke *et al*., 2015).

The issue of infertility and ARTs seems to be, at first sight, far from the field of public health and collective health, and nothing could be farther from the truth in this case. On the contrary, some issues show techno-scientific tendencies both in their practice and in reflections from different disciplines. They are part of the contemporary science and technology agenda, and from the bioethical and political viewpoint, a challenge concerning equity and justice.

The impossibility of having children has traditionally been seen as a private issue (Fidler & Bernstein, 1999). However, public health can contribute significantly by increasing knowledge about infertility, generating healthcare policies for the reasonable prevention of this condition, promoting access to medical care that could include ARTs, and, if doing so, requiring specific regulations. This is even reflected in the specialized academic publications, where a low representation of the issue of infertility is evident even in health journals that focus on women (Place *et al*., 2018).

Two arguments usually emerge when addressing the issue of infertility in less developed countries: overpopulation, and the limited resources destined for health care (which they suppose, could not prioritize the issue of infertility, associating it to ART costs; Ombelet *&* Goossens, 2017). One should not overlook that it has long been known that infertility affects globally much less developed countries than developed countries. While globally estimated at 10% of the world’s population, 80% of infertility is found in less developed countries (Álvarez-Díaz, 2011). Also, the psychological, social, and economic consequences are worse for infertile men and women in these countries (Rouchou, 2013).

At first, talking about infertility in less developed countries is indeed a huge challenge (Asemota & Klatsky, 2015). However, no one reasonably proposes that ARTs be provided for the entire population. A first approach, of totally health-related inspiration, is the prevention and timely diagnosis of conditions that generate infertility (Macaluso *et al*., 2010). For instance, sexually transmitted infections are among the leading causes of infertility, especially in women (Tsevat *et al*., 2017), but also in men (Gimenes *et al*., 2014). This is because if the healthcare system does not work properly, these infections may not be diagnosed on time, or are misdiagnosed, or it may be that they are diagnosed on time and correctly, but an erroneous, suboptimal treatment is indicated due to a shortage of system resources, or simply the population does not have the means to pay for the appropriate treatment.

Finally, there is another high-relevance issue: globalization has reached the field of medical care. Not all countries have the same resources, and within them, not the entire population has the same economic possibilities. Virtually all Latin American countries have medical services that provide ARTs (as already stated, they are mostly private). This causes the emergence of an increasingly studied event: the so-called “medical tourism” (Álvarez-Díaz, 2012), which in this field is called “reproductive tourism” (although it is also called “cross-border reproductive care”). This is the reality throughout Latin America, and was evidenced by discarding several questionnaires precisely because of this type of population. It is also an urgent call to bioethical and legal regulations for this event in all countries, since not doing so attaches risks to public health, problems for international adoptions, and possibility human, tissue, and cell trafficking (Shalev *et al*., 2016). The issue of the possible improper marketing and cell trafficking has already been raised for ART, concerning oocytes in general (Neri *et al*., 2016), or the investigation of techniques such as mitochondrial replacement (Dickenson, 2013). As far as embryos are concerned, the possibility of generating markets from the perspective of stem cells has already been raised (Kahn, 2001), and it has already been proposed as a possibility for Latin America in the field of ART for clinical purposes (Álvarez-Díaz, 2005).

This type of research can provide data to analyze from the perspectives of public healthcare and collective healthcare, not only the issue in the health-disease-care process, but also the way to address them, generating reflections and arguments that support the construction of public policies in general, and healthcare policies in particular. If the issue of infertility and ARTs is viewed from the perspective of human rights, the purely individualistic vision can be changed, since respecting sexual and reproductive rights means respecting human rights.
